# Association between the triglyceride-glucose index and carotid plaque progression in patients with type 2 diabetes mellitus: a retrospective cohort study

**DOI:** 10.3389/fendo.2025.1709079

**Published:** 2025-11-25

**Authors:** Zhangxiang Zhu, Xuan Xu, Guojuan Wang, Qianqian Zhang, Ying Li, Jun Zhu, Li Chen, Qibao Ye, Mingwei Chen

**Affiliations:** 1 Department of Endocrinology, First Affiliated Hospital of Anhui Medical University, Hefei, China; 2 Department of Endocrinology, The First People’s Hospital of Hefei, Hefei, China; 3 Department of Neurology, Hefei Eighth People’s Hospital, Hefei, China; 4 School of Clinical Medicine, Wannan Medical College, Wuhu, China

**Keywords:** T2DM, carotid plaque, TyG, insulin resistance, hyperinsulinemia

## Abstract

**Background:**

The triglyceride-glucose (TyG) index is a novel marker of insulin resistance associated with carotid vascular disease; however, its role in type 2 diabetes mellitus (T2DM) remains unclear.

**Methods:**

This retrospective study enrolled patients with T2DM who underwent repeated hospitalizations at our hospital between 2014 and 2024. Participants were stratified into tertiles based on TyG index values. Multivariable regression analyses were performed to assess the association between TyG index levels and carotid plaque progression.

**Results:**

A total of 548 patients with T2DM (55.93 ± 12.26 years, 60.4% males) were included, with a median follow-up time of 4 (2–7) years. The prevalence of carotid plaque progression increased stepwise with higher TyG tertiles (45.9% vs. 62.6% vs. 57.4%; p = 0.004), and the growth rates of bilateral plaque length and width also increased (all p < 0.05). The positive association between the TyG index and the growth rates of left carotid plaque length, left width, and right width remained significant in the fully adjusted model (TyG, per 1-unit increase: 0.197 mm vs. 0.196 mm vs. 0.189 mm; tertiles of TyG: 0.156 mm vs. 0.162 mm vs. 0.164 mm; all p < 0.05). After multivariable adjustment, Cox regression analysis showed that higher TyG index levels were associated with 1.261-fold, 1.244-fold, and 1.378-fold increased risks of carotid plaque progression in patients with T2DM (all p < 0.05). ROC curve analysis indicated that the TyG index exhibited modest predictive value for carotid plaque progression in patients with T2DM, with an AUC of 0.556 (p = 0.024). Adding the TyG index to the predictive model improved the C-statistic (0.624 vs. 0.649; p < 0.001), NRI = 0.211 (p = 0.015), and IDI = 0.020 (p < 0.001).

**Conclusion:**

In patients with T2DM, a higher TyG index is an independent risk factor for carotid plaque progression.

## Introduction

Insulin resistance, characterized by diminished efficiency of insulin in lowering blood glucose within the body, is one of the fundamental mechanisms underlying the pathogenesis of type 2 diabetes mellitus (T2DM) (1). In recent years, insulin resistance has also been recognized as a risk factor for vascular complications involving both macrovascular and microvascular diseases, and it contributes to the pathogenesis of diabetic nephropathy, retinopathy, and peripheral vascular disease in T2DM (2, 3).

The hyperinsulinemic–euglycemic clamp, which involves continuous infusion of insulin and glucose to maintain normal blood glucose and assess insulin sensitivity by the glucose infusion rate, is the gold standard for determining insulin resistance. However, this test is operationally complex and costly, making it impractical for routine clinical research. The homeostasis model assessment of insulin resistance (HOMA-IR) is one of the most widely used indicators in clinical practice, yet it remains inaccessible in many primary hospitals due to the inability to measure insulin levels. Therefore, the triglyceride–glucose (TyG) index, as a novel marker, can be widely applied in clinical settings (4).

Previous studies have shown that the TyG index has good specificity and sensitivity in evaluating insulin resistance (5). It may serve as a dose–response indicator for carotid plaque in Chinese populations (6). Additionally, it is capable of predicting adverse cardiovascular events in patients with diabetes and acute coronary syndrome (ACS) (7), and is an independent risk factor for in-stent restenosis (ISR) after stent implantation in ACS (8).

However, there is currently a lack of research on the predictive value of the TyG index for carotid plaque progression, particularly in T2DM populations. This study aims to investigate the association between the TyG index and carotid plaque progression in patients with T2DM.

## Methods

### Study population

A total of 2,542 patients with type 2 diabetes mellitus (T2DM) who had been hospitalized at least twice in the Department of Endocrinology of our hospital between 2014 and 2024 and underwent carotid ultrasound examination were initially screened. For patients with multiple hospitalizations, only the examination results from the hospitalization with the longest follow-up duration were adopted. After applying inclusion and exclusion criteria, 548 patients were ultimately enrolled.

The inclusion criteria were as follows: (1) meeting the diagnostic criteria for T2DM.

The exclusion criteria were as follows: (1) type 1 diabetes, gestational diabetes mellitus, latent autoimmune diabetes in adults (LADA), other specific types of diabetes, or acute diabetic complications such as diabetic ketoacidosis (DKA) and hyperosmolar hyperglycemic state (HHS); and (2) acute hepatic or renal insufficiency, infections, or other stress conditions.

### Survey and measurements

Basic demographic information, including gender, age, height, weight, disease duration, smoking history, and alcohol consumption history, was collected through medical history taking. Blood pressure was measured using an electronic sphygmomanometer in a standardized manner. After an 8–12 h fast, 4–5 mL of venous blood was collected in the morning and tested in our hospital’s Laboratory Department using a Siemens fully automated biochemical analyzer. Routine testing indicators included fasting blood glucose (FBG), triglycerides (TG), lipid profile, and liver and kidney function.

Carotid ultrasounds were performed by trained sonographers using a Toshiba (Japan) Aplio 500 color Doppler ultrasound device.

### Calculation formula and indicator definitions

Body mass index (BMI) = weight/height² (kg/m²). TyG index = ln[fasting TG (mg/dL) × FBG (mg/dL)/2] (5). Based on baseline TyG index tertiles, the subjects were divided into three groups: T1: (TyG ≤ 9.04), T2:(9.04 < TyG ≤ 9.72), and T3: (TyG > 9.72). Carotid plaque progression was defined as new carotid plaque development or an increase in plaque length or width exceeding 50% from baseline measurements.

### Statistical analysis

Statistical analyses were performed using SPSS 27.0. Continuous data are presented as mean±standard deviation or median and interquartile range, depending on the normality of the distribution. Comparisons of continuous variables across multiple groups were conducted using one-way ANOVA, while categorical variables were analyzed using variance tests.

Linear regression models were used to analyze the relationship between the TyG index and the growth rate of plaque length or width. Cox proportional hazards regression models were employed to explore the association between the TyG index and carotid plaque progression.

Model 1 was adjusted for gender and age. Model 2, based on Model 1, was further adjusted for BMI, diabetes duration, smoking history, alcohol consumption history, history of hypertension, and history of cerebral infarction. Model 3, based on Model 2, was additionally adjusted for total cholesterol (TC), low-density lipoprotein cholesterol (LDL-C), and high-density lipoprotein cholesterol (HDL-C).

The predictive value of the TyG index for carotid plaque progression was evaluated using ROC curves, with the area under the curve (AUC) and optimal cutoff value calculated. Furthermore, the C-statistic was computed and compared using DeLong’s test to determine whether incorporating the TyG index into the established risk factor model (including age, gender, BMI, disease duration, smoking history, history of hypertension, and history of statin use) improved predictive performance.

The net reclassification improvement (NRI) and integrated discrimination improvement (IDI) were calculated to further assess the incremental predictive value of the TyG index. A two-sided p-value < 0.05 was considered statistically significant.

## Results

### Baseline characteristics

As shown in **Table 1**, a total of 548 patients with T2DM were included in this study, with a median follow-up time of 4 (2–7) years. The cohort comprised 331 males (60.4%) and 217 females (39.6%), with a mean age of 55.93 ± 12.26 years. A total of 303 (55.3%) patients with T2DM experienced carotid plaque progression during the follow-up period.

**Table 1:** Baseline characteristics of patients stratified by tertile of TyG index.

Variables	Total(n=548)	TyG Tertile			P-value
		T1(n=183)	T2(n=182)	T3(n=183)	
TyG index	9.39±0.86	8.49±0.44	9.36±0.19	10.34±0.52	<0.001
Gender(male/%)	331/60.4	109/59.6	100/54.9	122/66.7	0.070
Age(years)	55.93±12.26	57.59±11.68	57.47±12.80	52.72±11.71	<0.001
Smoking History(n/%)	190/34.7	55/30.1	61/33.5	74/40.4	0.105
Alcohol History(n/%)	105/19.2	28/15.3	27/14.8	50/20.7	0.003
Hypertension History(n/%)	247/45.1	68/37.2	82/45.1	91/53.0	0.010
DM duration(years)	6(3,10)	7(3,10)	6(2,10)	5(3,10)	0.552
CI History(n/%)	60/10.9	18/9.8	29/15.9	13/7.1	0.022
Follow-up(years)	4(2,7)	5(3,7)	4(2.75,7)	4(2,7)	0.307
BMI(kg/m^2^)	24.61(22.34,26.98)	23.62(21.00,25.59)	24.21(22.07,26.18)	25.95(24.29,28.07)	<0.001
SBP(mmHg)	128(120,140)	120(110,138)	130(119.5,140)	130(120,140)	0.018
DBP(mmHg)	80(70,82)	78(70,80)	78(70,82)	80(74,85)	<0.001
Scr(umol/L)	64.10(51.90,75.90)	62.40(51.17,78.37)	61.40(50.30,73.30)	66.85(54.02,76.60)	0.106
UA(umol/L)	302.50±85.03	281.75±75.90	292.90±75.46	332.64±94.03	<0.001
FBG(mmol/L)	8.55(6.97,11.05)	6.94(5.79,8.18)	8.86(7.57,10.78)	10.67(8.56,12.98)	<0.001
TG(mmol/L)	1.63(1.03,2.68)	0.93(0.69,1.18)	1.62(1.26,12.03)	3.39(2.54,5.00)	<0.001
TC(mmol/L)	4.68±1.03	4.15±0.85	4.69±0.84	5.21±1.10	<0.001
LDL-C(mmol/L)	2.65±0.90	2.60±0.71	2.92±0.76	2.42±1.12	<0.001
HDL-C(mmol/L)	1.00(0.82,1.15)	1.07(0.92,1.31)	0.96(0.83,1.12)	0.91(0.79,1.08)	<0.001

TyG index, triglyceride-glucose index; DM, diabetes; CI, cerebral infarction; BMI, body mass index; SDP, systolic blood pressure; DBP, diastolic blood pressure; Scr, serum creatinine; UA, uric acid; FBG, fasting blood glucose; TG, triglycerides; TC, total cholesterol; LDL-C, low-density lipoprotein-C; HDL-C, high-density lipoprotein-C.

Patients were stratified into tertiles based on baseline triglyceride–glucose (TyG) index: T1 (TyG ≤ 9.04), T2 (9.04 < TyG ≤ 9.72), and T3 (TyG > 9.72). No significant differences were observed across the three groups in gender, smoking history, diabetes duration, follow-up duration, or serum creatinine (Scr). However, significant differences were noted in age, alcohol consumption history, hypertension history, cerebral infarction (CI) history, BMI, systolic blood pressure (SBP), diastolic blood pressure (DBP), uric acid (UA), fasting blood glucose (FBG), triglycerides (TG), total cholesterol (TC), low-density lipoprotein cholesterol (LDL-C), and high-density lipoprotein cholesterol (HDL-C) (**Table 1**).

### Carotid plaque data characteristics

**Figure 1:**
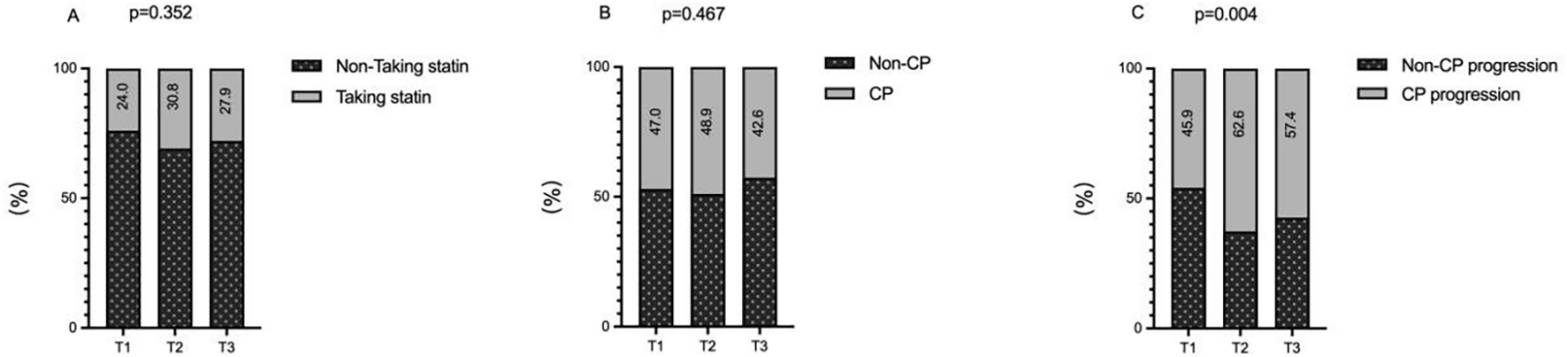
The impacts of the TyG index on the history of statin use **(A)**, prevalence of carotid plaque **(B)** and incidence of carotid plaque progression **(C)**. TyG index, triglyceride-glucose index; CP, carotid plaque.

There was no significant difference in the proportion of patients with a history of statin use at baseline across the three groups (24.0% vs. 30.8% vs. 27.9%; p = 0.352 > 0.05; **Figure 1A**). Baseline carotid plaque prevalence was 47.0%, 48.9%, and 42.6% in the three groups, respectively, with no statistical difference (p = 0.467 > 0.05; **Figure 1B**).

At the final follow-up, the rate of carotid plaque progression differed significantly across the groups, showing a rising trend with increasing TyG index: 45.9%, 62.6%, and 57.4% (p = 0.004 < 0.05; **Figure 1C**).

**Figure 2:**
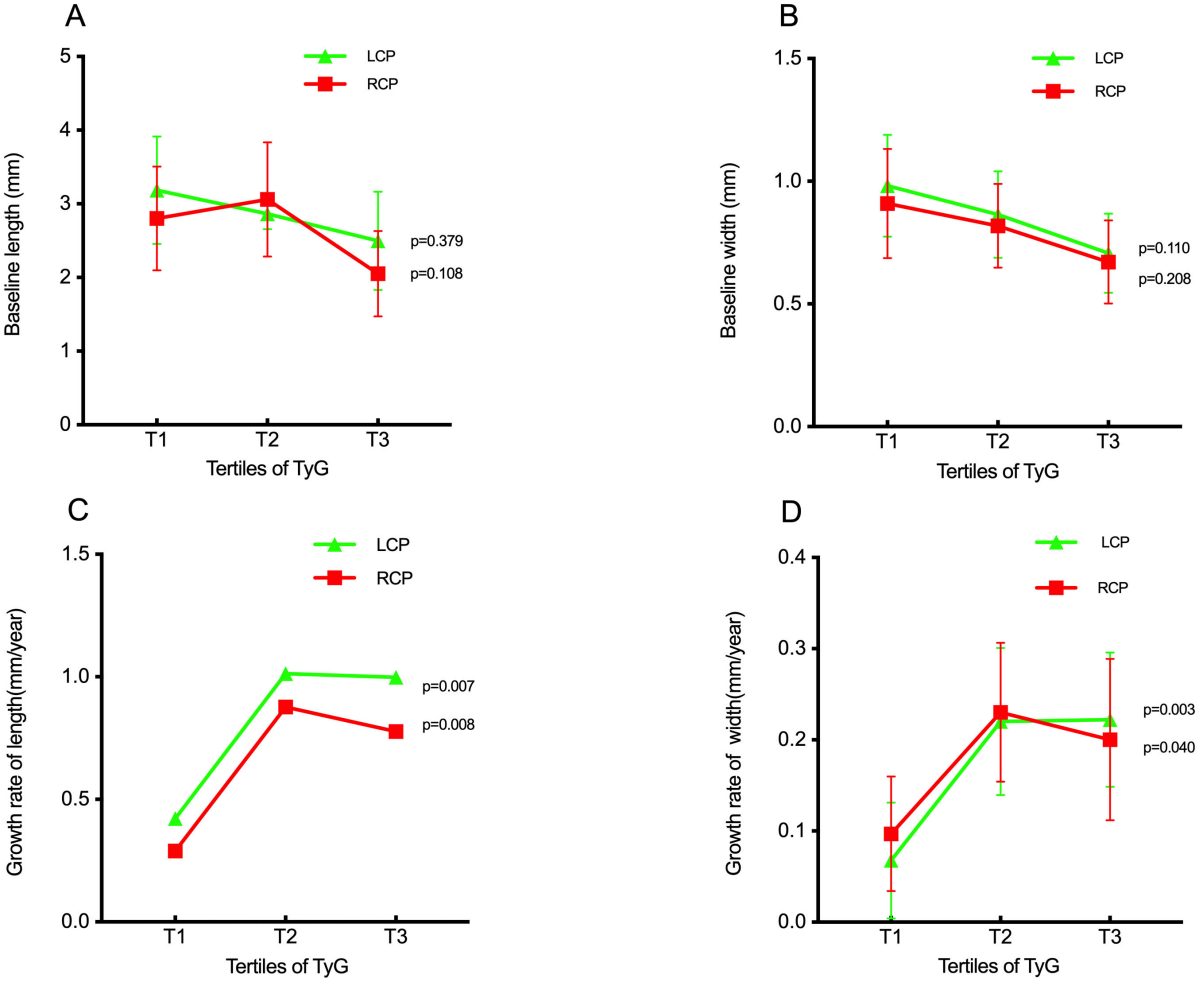
The impact of the TyG index on the baseline length of carotid plaque **(A)**, baseline width of carotid plaque **(B)**, growth rate of length of carotid plaque **(C)**, growth rate of width of carotid plaque **(D)**. TyG index, triglyceride-glucose index; LCP, left carotid plaque: RCP, right carotid plaque.

Baseline measurements of bilateral carotid plaque length and width showed minor differences across the three groups, with all p-values > 0.05, indicating no statistical significance (**Figures 2A, B**). In contrast, the growth rates of bilateral carotid plaque length and width differed significantly among the groups (all p < 0.05), with these rates accelerating overall as the TyG index increased (**Figures 2C, D**).

### Association of TyG index with carotid plaque growth rate in multiple logistic regression models

Multiple linear regression analysis revealed that in Model 3—adjusted for age, gender, smoking history, alcohol consumption, hypertension, cerebral infarction, BMI, disease duration, TG, TC, LDL-C, HDL-C, and statin use—when the TyG index was treated as a continuous variable, each 1-unit increase was associated with significant increases in the annual growth rates of left carotid plaque length (0.197 mm), left carotid plaque width (0.196 mm), and right carotid plaque width (0.189 mm; all p < 0.05; **Table 2A**).

**Table 2A:** Association of TyG index with carotid plaque growth rate in multiple logistic regression models.

Growth Rate	TyG as continuous variable								
	Model1			Model2			Model3		
	β	95%CI	P	β	95%CI	P	β	95%CI	P
Length of LCP	0.140	0.130 to 0.547	0.002	0.144	0.126 to 0.571	0.002	0.197	0.089 to 0.861	0.016
Width of LCP	0.131	0.025 to 0.124	0.003	0.115	0.013 to 0.118	0.015	0.196	0.019 to 0.202	0.018
Length of RCP	0.093	0.011 to 0.378	0.038	0.099	0.011 to 0.413	0.038	0.079	-0.176 to 0.503	0.343
Width of RCP	0.061	-0.016 to 0.090	0.173	0.073	-0.012 to 0.101	0.126	0.189	0.016 to 0.211	0.022

When analyzed as a categorical variable, a higher TyG index was linked to a gradual increase in the risk of elevated annual growth rates for left carotid plaque length, left carotid plaque width, and right carotid plaque width (all p < 0.05; **Table 2B**). Notably, regardless of whether the TyG index was treated as a continuous or categorical variable, no statistically significant difference was observed in the annual growth rate of right carotid plaque length across groups (all p > 0.05; **Tables 2A**, **B**).

**Table 2B:** Association of TyG index as a categorical variable with carotid plaque growth rate in multiple logistic regression models.

Growth Rate	TyG as categorical variable								
	Model1			Model2			Model3		
	β	95%CI	P	β	95%CI	P	β	95%CI	P
Length of LCP	0.149	0.162 to 0.595	<0.001	0.157	0.166 to 0.626	<0.001	0.156	0.092 to 0.697	0.011
Width of LCP	0.145	0.035 to 0.137	0.001	0.133	0.025 to 0.133	0.004	0.162	0.024 to 0.168	0.009
Length of RCP	0.122	0.076 to 0.456	0.006	0.131	0.084 to 0.488	0.006	0.121	-0.001 to 0.531	0.051
Width of RCP	0.085	-0.002 to 0.108	0.058	0.100	0.005 to 0.122	0.034	0.164	0.027 to 0.180	0.008

Model 1: adjusted for age, sex.

Model 2: adjusted for age, sex, BMI, course of DM and history of smoking, alcohol, hypertension, CI.

Model 3: adjusted for age, sex, BMI, course of DM and history of smoking, alcohol, hypertension, CI and TC, LDL-C, HDL-C and history of taking statin.

TyG index, triglyceride-glucose index; BMI, body mass index; CI, cerebral infarction; DM, diabetes; TC, total cholesterol; LDL-C, low-density lipoprotein-C; HDL-C, high-density lipoprotein-C.

### Association of TyG index with carotid plaque progressed in cox proportional hazard model analysis

Cox proportional hazards regression models showed that, after adjusting for confounding factors in Models 1, 2, and 3, the risk of carotid plaque progression in patients with T2DM increased with a higher TyG index, with hazard ratios (HRs) of 1.261, 1.244, and 1.378, respectively (all p < 0.05; **Table 3**).

**Table 3:** Association of TyG index with carotid plaque progressed in Cox proportional hazard model analysis.

Model	β	SE	Wald	P	HR	95%CI
Model1	0.232	0.072	10.335	0.001	1.261	1.095 to 1.453
Model2	0.218	0.076	8.129	0.004	1.244	1.071 to 1.445
Model3	0.32	0.147	4.778	0.029	1.378	1.034 to 1.836

Model 1: adjusted for age and sex.

Model 2: adjusted for age, sex, BMI, duration of DM, and history of smoking, alcohol consumption, hypertension, and CI.

Model 3: adjusted for age, sex, BMI, duration of DM, and history of smoking, alcohol consumption, hypertension, CI and TC, LDL-C, HDL-C, and statin use.

TyG index, triglyceride-glucose index; BMI, body mass index; CI, cerebral infarction; DM, diabetes; TC, total cholesterol; LDL-C, low-density lipoprotein-C; HDL-C, high-density lipoprotein-C.

### Incremental effects of the TyG index on the predictive value of carotid plaque progressed

**Figure 3:**
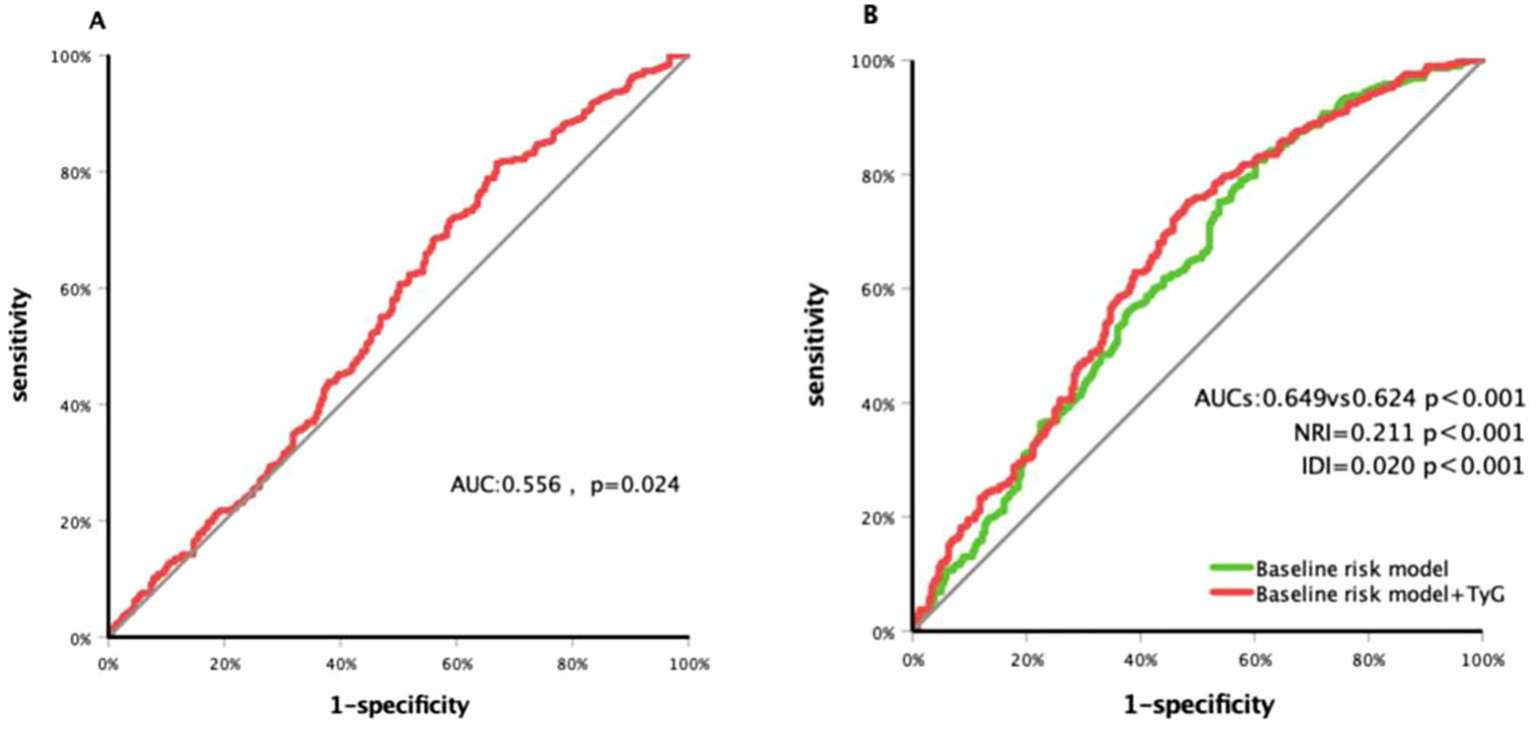
Receiver operating characteristic curve analysis of the TyG index to predict carotid plaque progressed **(A)** and comparison of the G-statistics between the models **(B)**. Baseline risk model conclude age, sex, duration of DM and history of smoking, hypertension and taking statin; TyG index, triglyceride-glucose index; DM, diabetes; AUC, area under curve; NRI, net reclassification improvement; IDI, integrated discrimination improvement.

ROC curve analysis (**Figure 3A**) showed that the TyG index had modest predictive value for carotid plaque progression in patients with T2DM, with an area under the curve (AUC) of 0.556 (95% CI: 0.507–0.605; p = 0.024) and an optimal cutoff value of 8.86 (sensitivity: 81.5%; specificity: 33.1%).

Meanwhile, a risk model incorporating age, gender, BMI, diabetes duration, smoking history, hypertension history, and statin use history yielded a C-statistic of 0.624 (95% CI: 0.576–0.673; p < 0.001; **Figure 3B**). After adding the TyG index, the C-statistic increased to 0.649 (95% CI: 0.602–0.697; p < 0.001), with a net reclassification improvement (NRI) of 0.211 (95% CI: 0.041–0.382; p = 0.015) and an integrated discrimination improvement (IDI) of 0.020 (95% CI: 0.000–0.032; p < 0.001).

## Discussion

The triglyceride–glucose (TyG) index, a novel marker for assessing insulin resistance, is widely adopted in primary care owing to its simplicity, cost-effectiveness, and reproducibility. Its applications include community-based cardiovascular risk screening, early identification of metabolic syndrome and type 2 diabetes mellitus (T2DM), and prognostic evaluation of atherosclerotic diseases.

Prior studies have identified a positive correlation between the TyG index and multiple carotid plaques in hypertensive populations populations (9). In non-diabetic cohorts, an elevated TyG index is associated with a significantly higher prevalence of carotid plaques and unstable carotid plaques plaques (10, 11). Some research indicates that the TyG index outperforms HOMA-IR in predictive capacity (12), particularly in overweight/obese individuals and those with impaired glucose tolerance or diabetes (13). A single-center retrospective study from Suzhou Municipal Hospital reported a significant association between the TyG index and the risk of unstable carotid plaques, with the strongest correlation observed in diabetic populations populations (14) Additionally, studies have demonstrated that in individuals with prediabetes, arteriosclerosis is independently associated with triglycerides/high-density lipoprotein (TG/HDL), whereas no association with the TyG index has been observed (15).However, research on the TyG index and carotid plaques specifically in T2DM populations remains limited. Thus, this single-center retrospective observational cohort study in patients with T2DM aimed to investigate the potential association between the TyG index and the risk of carotid plaque progression and to evaluate the TyG index’s predictive value for such progression.

Our results showed that when stratified into TyG tertiles, baseline carotid plaque length and width did not differ significantly across the three groups. However, the annual growth rates of plaque length and width increased with higher TyG index. Multiple linear regression analysis—fully adjusted for age, gender, smoking, alcohol consumption, hypertension, cerebral infarction, BMI, disease duration, triglycerides (TG), total cholesterol (TC), low-density lipoprotein cholesterol (LDL-C), high-density lipoprotein cholesterol (HDL-C), and statin use—revealed that a higher TyG index, whether analyzed as a continuous or categorical variable, was associated with increased annual growth rates of left carotid plaque length, left carotid plaque width, and right carotid plaque width.

Pan et al (10). reported in a cross-sectional study that coal miners with a high TyG index had a higher prevalence of carotid plaques. Bosco et al. (16) demonstrated that in patients with familial hypercholesterolemia, the subclinical atherosclerosis group had a higher TyG index compared with the non-subclinical atherosclerosis group— which indicates that a higher TyG index is typically associated with a relatively higher prevalence of subclinical atherosclerosis. Unlike their findings, our study did not detect significant differences in carotid plaque prevalence across TyG tertiles in T2DM patients, potentially due to variations in study populations. Zhang et al. (6), in a 4-year follow-up of 2,300 individuals undergoing physical examinations, found that a higher TyG index correlated with increased risk of new carotid plaque development. Despite differing study populations, their conclusions align with our observation that patients in the higher TyG groups exhibited a greater incidence of carotid plaque progression. Cox proportional hazards regression models further confirmed that a higher TyG index was associated with an increased risk of carotid plaque progression in T2DM patients; after adjusting for multiple confounders, Model 3 showed a 37.8% elevated risk of progression.

Most existing studies indicate that the TyG index has mild to moderate predictive value for carotid plaques (17, 18). Consistently, some research has shown that incorporating the TyG index into cardiovascular risk prediction models improves their performance (19). In our study, ROC curve analysis demonstrated modest predictive value of the TyG index for carotid plaque progression in patients with T2DM. However, adding the TyG index to an established risk prediction model enhanced its ability to predict plaque progression risk, suggesting that the TyG index could serve as a valuable component in cardio-cerebrovascular risk prediction models, thereby improving their accuracy.

Recently, the triglyceride–glucose (TyG) index has also been employed as a marker of insulin resistance in a study focusing on patients with heterozygous familial hypercholesterolemia (20). This is because the TyG index is calculated based on fasting blood glucose and triglyceride levels, and its elevation reflects the coexistence of glucotoxicity and lipotoxicity. Scholars have proposed that glucose and lipid metabolism disorders can induce vascular injury through shared pathological and molecular mechanisms, including endothelial dysfunction, advanced glycation end-product formation, oxidative stress, chronic inflammation, epigenetic modification, and other processes (21). Glucolipotoxicity induces pancreatic β-cell apoptosis and dysfunction via endoplasmic reticulum stress, mitochondrial impairment, defective autophagy, and inflammation, ultimately contributing to insulin resistance (22). Insulin resistance, in turn, is a known risk factor for atherosclerosis: it impairs the phosphatidylinositol 3-kinase–nitric oxide pathway in endothelial cells, activates the mitogen-activated protein kinase pathway (promoting vasoconstriction), and directly stimulates vascular smooth muscle cells to induce vasoconstriction independently of endothelial cells (23).

Additionally, in the context of insulin resistance, glucolipotoxicity can initiate autophagy while inhibiting autophagic flux via the adenylate-activated protein kinase (AMPK) and mammalian target of rapamycin (mTOR) signaling pathways, leading to autophagosome accumulation and endothelial dysfunction20. Insulin resistance also activates the mitochondrial electron transport chain, inducing overproduction of reactive oxygen species (ROS) and causing endothelial damage (24). Furthermore, excess free fatty acids generated during insulin resistance contribute to atherogenesis (25). Concurrently, insulin resistance reduces the number of vascular endothelial progenitor cells, impairing endothelial self-repair and promoting atherosclerosis and plaque formation (26). Hyperinsulinemia is a pathological state associated with insulin resistance. When insulin resistance occurs in the body, the pancreas secretes excessive insulin compensatorily, resulting in hyperinsulinemia. This chronic hyperinsulinemia exposes the vascular endothelium to a high-insulin environment for a prolonged period, downregulates the PI3K/Akt/eNOS signaling axis, reduces nitric oxide (NO) production, and simultaneously enhances the pro-inflammatory and adhesive functions of the endothelium, ultimately leading to vascular endothelial dysfunction—the initial link in the development of atherosclerosis (27). In addition, hyperinsulinemia can activate the INSR/IRS/PI3K/Akt/mTOR signaling pathway, promoting inhibition of lipolysis and enhancement of lipogenesis in adipocytes, while lipid metabolism disorders remain the core driving factor for lipid accumulation in atherosclerosis (28). Therefore, in the prevention of atherosclerosis, patients with T2DM need to pay attention to whether blood lipid levels meet target values while maintaining glycemic control, particularly the level of LDL-C. However, regarding the current status of lipid-lowering therapy, the LDL-C target achievement rate among high- and very-high-risk patients (e.g., those with atherosclerotic cardiovascular disease, diabetes mellitus, or familial hypercholesterolemia) remains extremely low. In recent years, inclisiran—a novel small interfering RNA (siRNA) drug and the first agent targeting PCSK9—has provided an excellent solution to this challenge (29). It is hoped that such drugs will eventually become as accessible and easy to use as the TyG index and will be popularized in primary community hospitals.

## Conclusion

In conclusion, this study demonstrates that a higher TyG index is associated with carotid plaque progression in patients with T2DM, serving as a convenient, practical, and reliable evaluation indicator for predicting carotid plaque progression in this population. Whether active early intervention to control blood glucose and lipids in patients with T2DM and a high TyG index can effectively delay carotid plaque progression requires further validation through prospective studies.

## Data Availability

The raw data supporting the conclusions of this article will be made available by the authors, without undue reservation.

## References

[1] Cho H Lai CC Bonnavion R Alnouri MW Wang S Roquid KA . Endothelial insulin resistance induced by adrenomedullin mediates obesity-associated diabetes. Science. (2025) 387:674–82. doi: 10.1126/science.adr4731, PMID: 39913566

[2] Naja K Anwardeen N Albagha O Elrayess MA . Lipid subclasses differentiate insulin resistance by triglyceride-glucose index. Metabolites. (2025) 15:342. doi: 10.3390/metabo15050342, PMID: 40422918 PMC12113954

[3] Li HF Miao X Li Y . The triglyceride glucose (TyG) index as a sensible marker for identifying insulin resistance and predicting diabetic kidney disease. Med Sci Monit. (2023) 29:e939482. doi: 10.12659/MSM.939482, PMID: 37421131 PMC10337482

[4] Adams-Huet B Zubirán R Remaley AT Jialal I . The triglyceride-glucose index is superior to homeostasis model assessment of insulin resistance in predicting metabolic syndrome in an adult population in the United States. J Clin Lipidol. (2024) 18:e518–24. doi: 10.1016/j.jacl.2024.04.130, PMID: 38834412

[5] Guerrero-Romero F Simental-Mendía LE González-Ortiz M Martínez-Abundis E Ramos-Zavala MG Hernández-González SO . The product of triglycerides and glucose, a simple measure of insulin sensitivity. Comparison with the euglycemic-hyperinsulinemic clamp. J Clin Endocrinol Metab. (2010) 95:3347–51. doi: 10.1210/jc.2010-0288, PMID: 20484475

[6] Zhang Y Wu Z Li X Wei J Zhang Q Wang J . Association between the triglyceride-glucose index and carotid plaque incidence: a longitudinal study. Cardiovasc Diabetol. (2022) 21:244. doi: 10.1186/s12933-022-01683-6, PMID: 36380351 PMC9667568

[7] Wang L Cong HL Zhang JX Hu YC Wei A Zhang YY . Triglyceride-glucose index predicts adverse cardiovascular events in patients with diabetes and acute coronary syndrome. Cardiovasc Diabetol. (2020) 19:80. doi: 10.1186/s12933-020-01054-z, PMID: 32534586 PMC7293784

[8] Zhu Y Liu K Chen M Liu Y Gao A Hu C . Triglyceride-glucose index is associated with in-stent restenosis in patients with acute coronary syndrome after percutaneous coronary intervention with drug-eluting stents. Cardiovasc Diabetol. (2021) 20:137. doi: 10.1186/s12933-021-01332-4, PMID: 34238294 PMC8268452

[9] Liu S Zhang H Wu M Zhou Z Xiao Y Wan Q . Association between the triglyceride-glucose index and carotid artery plaque burden in patients with primary hypertension: A cross-sectional study. Clin Exp Hypertens. (2024) 46:2383232. doi: 10.1080/10641963.2024.2383232, PMID: 39045803

[10] Pan J Yang B Wang Z Tang L Jia P Yang S . Triglyceride-glucose index is related to carotid artery plaque in railway workers: A cross-sectional study. Diabetes Metab Syndr Obes. (2023) 16:2561–71. doi: 10.2147/DMSO.S418358, PMID: 37645236 PMC10461744

[11] Wang A Tian X Zuo Y Zhang X Wu S Zhao X . Association between the triglyceride-glucose index and carotid plaque stability in nondiabetic adults. Nutr Metab Cardiovasc Dis. (2021) 31:2921–8. doi: 10.1016/j.numecd.2021.06.019, PMID: 34353702

[12] Minh HV Tien HA Sinh CT Thang DC Chen CH Tay JC . Assessment of preferred methods to measure insulin resistance in Asian patients with hypertension. J Clin Hypertens (Greenwich). (2021) 23:529–37. doi: 10.1111/jch.14155, PMID: 33415834 PMC8029536

[13] Vasques AC Novaes FS de Oliveira Mda S Souza JR Yamanaka A Pareja JC . TyG index performs better than HOMA in a Brazilian population: a hyperglycemic clamp validated study. Diabetes Res Clin Pract. (2011) 93:e98–e100. doi: 10.1016/j.diabres.2011.05.030, PMID: 21665314

[14] Huo G Zheng J Cao J Zhang L Yao Z Zeng Y . Association between triglyceride-glucose index and carotid plaque stability in different glycemic status: A single-center retrospective study. J Am Heart Assoc. (2025) 14:e037970. doi: 10.1161/JAHA.124.037970, PMID: 39846306 PMC12074782

[15] Di Marco M Scilletta S Miano N Capuccio S Musmeci M Di Mauro S . Triglycerides to high density lipoprotein cholesterol ratio (TG/HDL), but not triglycerides and glucose product (TyG) index, is associated with arterial stiffness in prediabetes. Diabetes Res Clin Pract. (2025) 224:112189. doi: 10.1016/j.diabres.2025.112189, PMID: 40252776

[16] Bosco G Di Giacomo Barbagallo F Di Marco M Scilletta S Miano N Capuccio S . Evaluations of metabolic and innate immunity profiles in subjects with familial hypercholesterolemia with or without subclinical atherosclerosis. Eur J Intern Med. (2025) 132:118–26. doi: 10.1016/j.ejim.2024.12.002, PMID: 39672731

[17] Li J Dong Z Wu H Liu Y Chen Y Li S . The triglyceride-glucose index is associated with atherosclerosis in patients with symptomatic coronary artery disease, regardless of diabetes mellitus and hyperlipidaemia. Cardiovasc Diabetol. (2023) 22:224. doi: 10.1186/s12933-023-01919-z, PMID: 37620954 PMC10463708

[18] Moon JH Kim Y Oh TJ Moon JH Kwak SH Park KS . Triglyceride-glucose index predicts future atherosclerotic cardiovascular diseases: A 16-year follow-up in a prospective, community-dwelling cohort study. Endocrinol Metab (Seoul). (2023) 38:406–17. doi: 10.3803/EnM.2023.1703, PMID: 37533176 PMC10475965

[19] Wu X Qiu W Yang H Chen YJ Liu J Zhao G . Associations of the triglyceride-glucose index and atherogenic index of plasma with the severity of new-onset coronary artery disease in different glucose metabolic states. Cardiovasc Diabetol. (2024) 23:76. doi: 10.1186/s12933-024-02163-9, PMID: 38378553 PMC10880297

[20] González-Lleó AM Brito-Casillas Y Martín-Santana V Gil-Quintana Y Tugores A Scicali R . Glucose metabolism in heterozygous familial hypercholesterolemia with a founder effect and a high diabetes prevalence: a cross-sectional study. Cardiovasc Diabetol. (2025) 24:322. doi: 10.1186/s12933-025-02857-8, PMID: 40770647 PMC12330011

[21] Chen X Shi C Wang Y Yu H Zhang Y Zhang J . The mechanisms of glycolipid metabolism disorder on vascular injury in type 2 diabetes. Front Physiol. (2022) 13:952445. doi: 10.3389/fphys.2022.952445, PMID: 36117707 PMC9473659

[22] Lytrivi M Castell AL Poitout V Cnop M . Recent insights into mechanisms of β-cell lipo- and glucolipotoxicity in type 2 diabetes. J Mol Biol. (2020) 432:1514–34. doi: 10.1016/j.jmb.2019.09.016, PMID: 31628942 PMC7073302

[23] Artunc F Schleicher E Weigert C Fritsche A Stefan N Häring HU . The impact of insulin resistance on the kidney and vasculature. Nat Rev Nephrol. (2016) 12:721–37. doi: 10.1038/nrneph.2016.145, PMID: 27748389

[24] Liu Y Xiang H Xiong W Ouyang J Liu H Zhao S . Glucolipotoxicity induces endothelial cell dysfunction by activating autophagy and inhibiting autophagic flow. Diabetes Vasc Dis Res. (2022) 19:14791641221102513. doi: 10.1177/14791641221102513, PMID: 35549572 PMC9125420

[25] Cubbon RM Kahn MB Wheatcroft SB . Effects of insulin resistance on endothelial progenitor cells and vascular repair. Clin Sci (Lond). (2009) 117:173–90. doi: 10.1042/CS20080263, PMID: 19630751

[26] Humpert PM Djuric Z Zeuge U Oikonomou D Seregin Y Laine K . Insulin stimulates the clonogenic potential of angiogenic endothelial progenitor cells by IGF-1 receptor-dependent signaling. Mol Med. (2008) 14:301–8. doi: 10.2119/2007-00052.Humpert, PMID: 18309377 PMC2255559

[27] Doronzo G Viretto M Russo I Mattiello L Di Martino L Cavalot F . Nitric oxide activates PI3-K and MAPK signalling pathways in human and rat vascular smooth muscle cells: influence of insulin resistance and oxidative stress. Atherosclerosis. (2011) 216:44–53. doi: 10.1016/j.atherosclerosis.2011.01.019, PMID: 21316056

[28] Zhang AMY Wellberg EA Kopp JL Johnson JD . Hyperinsulinemia in obesity, inflammation, and cancer. Diabetes Metab J. (2021) 45:285–311. doi: 10.4093/dmj.2020.0250, PMID: 33775061 PMC8164941

[29] Inclisiran . Reasons for a novel agent in a crowded therapeutic field. Curr Atheroscler Rep. (2025) 27:25. doi: 10.1007/s11883-024-01271-x, PMID: 39786678 PMC11717820

